# Constipation

**Published:** 1876-12

**Authors:** 


### CONSTIPATION.

In the editor's book on Health and Disease—the third edition of which was called for within nine months after its first issue—is an article on this subject, which ought to be read and studied, and practically observed from early childhood by every human being. No person can be well long who does not have one action of the bowels every twenty-four hours, and to maintain a healthful regularity by means of natural agencies, such as by the ordinary food and drink is of the utmost importance to all.

One bound volume of HALL'S JOURNAL OF HEALTH for one year, from 1854 to 1872 inclusive, will be sent by mail, postage paid, on receipt of sixty cents. Each volume is complete in itself, and is worth three times the priee demanded. Address, HALL'S JOURNAL OF HEALTH, 137 Eighth Street, New York.

